# A unified science of concussion

**DOI:** 10.1111/j.1749-6632.2010.05695.x

**Published:** 2010-10

**Authors:** Jun Maruta, Stephanie W Lee, Emily F Jacobs, Jamshid Ghajar

**Affiliations:** 1 Brain Trauma FoundationNew York, New York; 2 Department of Neurological Surgery, Weill-Cornell Medical CollegeNew York, New York

**Keywords:** smooth pursuit, attention, prefrontal cortex, diffuse axonal injury (DAI), blast injury

## Abstract

The etiology, imaging, and behavioral assessment of mild traumatic brain injury (mTBI) are daunting fields, given the lack of a cohesive neurobiological explanation for the observed cognitive deficits seen following mTBI. Although subjective patient self-report is the leading method of diagnosing mTBI, current scientific evidence suggests that quantitative measures of predictive timing, such as visual tracking, could be a useful adjunct to guide the assessment of attention and to screen for advanced brain imaging. Magnetic resonance diffusion tensor imaging (DTI) has demonstrated that mTBI is associated with widespread microstructural changes that include those in the frontal white matter tracts. Deficits observed during predictive visual tracking correlate with DTI findings that show lesions localized in neural pathways subserving the cognitive functions often disrupted in mTBI. Unifying the anatomical and behavioral approaches, the emerging evidence supports an explanation for mTBI that the observed cognitive impairments are a result of predictive timing deficits caused by shearing injuries in the frontal white matter tracts.

## Introduction

Cognitive sequelae from concussion, or mild traumatic brain injury (mTBI), are difficult to measure and often ascribed to the traumatic event or premorbid factors.^1,2^ Because computer tomography (CT) images are normal for most mTBI patients,^3^ little or no physical brain injury may be presumed^4^; however, the magnetic resonance imaging (MRI) technique of diffusion tensor imaging (DTI) can now detect microscopic brain white matter tract lesions.^5–8^ These lesions are likely to be responsible for the postconcussive symptoms and may explain chronic difficulties experienced by some patients.

Considering the vulnerability of anterior white matter tracts to shearing and the involvement of these tracts in attention and moment-to-moment predictive timing, it may be timely to develop a unified approach to the prevention, diagnosis, and treatment of mTBI.

### Incidence and definition

TBI has been referred to as the signature injury of the wars in Iraq and Afghanistan.^9,10^ An estimated 320,000 service members deployed between 2001 and 2007 screened positive for a probable TBI.^10^ Blast exposure has been indicated as the greatest source of injury accounting for the majority of TBIs sustained by service members.^11,12^ TBI is graded in degree, from mild to severe, based on the acute effects of the injury on an individual's level of arousal and duration of amnesia. The Veterans Affairs/Department of Defense Clinical Practice Guideline classifies mTBI as a traumatically induced structural injury or physiological disruption of brain function as a result of an external force, with normal CT structural imaging, loss of consciousness <30 min, alteration of mental state <24 h, posttraumatic amnesia <1 day, and Glasgow Coma Score of 13–15.^13^ A similar classification is used in the civilian population. The majority of TBIs sustained in both the military and civilian populations are classified as mild.^14,15^

Following mTBI, individuals can develop postconcussive syndrome (PCS): a constellation of symptoms that can be categorized as cognitive, affective, or somatic (Table 1).^16^ PCS may lead to chronic disability.^17,18^

**Table 1:** Postconcussive symptoms

Cognitive	Somatic	Affective
• Memory difficulties	• Headache	• Irritability
	• Dizziness	• Depression
• Decreased concentration	• Nausea	• Anxiety
	• Fatigue	
• Decreased processing speed	• Sleep disturbances	
	• Blurred vision	
	• Tinnitus	
	• Hypersensitivity to light or noise	

### Etiology and mechanism of injury

A common pathological feature of TBI includes distributed injuries to the subcortical white matter, or diffuse axonal injury (DAI), that may occur with or without a focal injury.^19–23^ mTBI may involve DAI.^24^ DAI presents, histologically, as microscopic lesions, myelin loss, axonal degeneration, or axonal swellings^19–23,25^ but is difficult to detect with traditional CT and MRI scans.^5,26–29^ In blunt closed-head injury, these diffuse axonal damages have been attributed to shear strain and tissue deformation caused by the rotational accelerations of the brain as an external force is applied to the head.^30,31^ The shear strains and tissue deformations of the primary biomechanical injury and reactive edema represent the acute phase of TBI. Acute TBI may lead to axonal degeneration and neuronal cell death (secondary injury) that develops after the initial biomechanical incident, which represents the chronic phase of TBI. The rotational acceleration experienced by the brain can be produced by either a linear or angular acceleration of the head because the brain's motion is constrained by basal-frontal tethering.^31^ It is of note that rotational acceleration of the brain, and thereby DAI, can be produced with or without a direct blow to the head as in cases of whiplash in a car accident.

Blast-related injuries can occur though a combination of four different mechanisms: primary (direct effects of the over- and under-pressure wave); secondary (effects of projectiles); tertiary (effects of wind, fragmentation of buildings and vehicles); and quaternary (burns, asphyxia, and exposure to toxic inhalants).^32^ The pathophysiology of blast-related TBI is complex and not fully understood. Although rotation-induced shearing is consistent with the secondary and tertiary effects, the primary effect alone is likely able to induce axonal injury.^33–35^ Regardless, the functional deficits associated with blast-related mTBI do not appear different from non-blast-related mTBI.^36,37^

## Current diagnostic methods in mTBI

### Military mTBI screening methods

Because the severity of TBI is defined by the acute injury characteristics, the term “mild” should not be interpreted as an indicator of PCS symptom severity; PCS may develop in the days following concussion, and the extent of disability and treatment needs vary from patient to patient.^17,18,38,39^ Currently, the method of mTBI diagnosis is highly dependent upon information obtained through patients’ subjective self-report about the acute characteristics of their injury. Unlike moderate or severe TBI, which are more easily diagnosed acutely by decrements in arousal or abnormality in CT images, mTBI is much more ambiguous during the acute phases and may not be diagnosed until the affected individual complains of postconcussive symptoms or experiences difficulties in their social interactions or in job or school performance. Adding to the complexity, as a consequence of cognitive impairments that result from their injury, mTBI patients may have a reduced awareness of their deficits.^4,40^ This is also the case with patients who have survived more severe TBIs.^41,42^ Because of these challenges, the Department of Defense and the Department of Veterans Affairs have implemented system-wide multipoint screening and assessment procedures for detecting mTBI in service members engaged in and returning from the wars in Iraq and Afghanistan.^13,43–45^

During deployment, the military administers the Military Acute Concussion Evaluation (MACE), an adaptation of Standardized Assessment of Concussion,^46^ as soon as possible following the injury. Postdeployment, the Brief Traumatic Brain Injury Survey (BTBIS)^47^ included in the Warrior Administered Retrospective Casualty Assessment Tool (WARCAT) or the Post-Deployment Health Assessment (PDHA) is administered to the soldiers.^48^ These screening measures are designed to be overly inclusive to reduce the risk of overlooking individuals with TBI;^44^ any positive screen would need to be followed by a clinical interview and examination to either confirm or negate the diagnosis of mTBI. Evidence of structural brain damage is not part of the mTBI diagnostic criteria.^13^

### Clinical assessments

Various methods exist to evaluate mTBI. Currently, neuropsychological testing is considered to be one of the most important assessment tools during both the acute and chronic phases of PCS. Typical neuropsychological batteries assess attention, working memory, and executive functions. One such battery, the Automated Neuropsychological Assessment Metric (ANAM), includes tasks like simple reaction time, code substitution, mathematical processing, and matching to sample.^49^ ANAM is administered to every service member prior to deployment, and changes in cognitive functions after an injury may be identified or monitored using this assessment tool. Neuropsychological tests are sensitive to moderate to severe TBI^1,2^ and may provide important insights into cognitive functioning during the acute phase of mTBI.^49,50^ However, neuropsychological test performance seems to return to normal within several months in the mTBI population at large^1,2,50^ and there is no association with number of lifetime TBIs, severity of TBI or number of postconcussive symptoms.^51^ Underlying causes of persistent cognitive difficulties are not clear, but it is possible that deficits are too subtle or not detectable by traditional neuropsychological testing methods.^18,50^

CT plays a critical role in the clinical management of TBI owing to its wide availability and its speed and accuracy in the detection of skull fractures and intracranial hemorrhage.^3,24,52^ CT is particularly useful for conditions that require immediate intervention and is indicated for moderate and severe TBI patients. However, CT performs poorly at detecting DAI,^5,26,27^ and images often present as normal for most mTBI patients.^3^

Standard structural MRI outperforms CT in detecting DAI and secondary lesions,^21,26^ and is often used in assessments of subacute and chronic TBI. However, DAI is still difficult to detect by conventional MRI,^5,27,28^ and the presence of pathology may not be detected in cases of mTBI.^29,53^ Neither CT nor MRI scans correlate well with the number of self-reported symptoms or performance on neuropsychological tests.^53–55^

Supplementing behavioral and structural assessment techniques, functional imaging can be used to evaluate the pathophysiological and functional sequelae of mTBI.^56^ These methods include functional MRI, positron emission tomography (PET), single photon emission computed tomography (SPECT), electroencephalography (EEG), and magnetoencephalography (MEG). Functional MRI may be used to assess the degree of neural activation in TBI subjects carrying out cognitive tasks known to be disrupted by TBI.^57^ Several PET studies show significant correlations between cognitive task performance and metabolic abnormalities^58–60^; however, the interpretations remain inconclusive regarding the types of metabolic changes in specific regions of interest across patients^59^ and the relationship between global abnormalities and specific cognitive tasks.^60^ SPECT may be able to predict neuropsychological test performance^61^ but results are inconsistent.^62^ Standard clinical EEG procedures used in hospitals detect abnormal activities caused by larger morphological changes like focal lesions and are therefore less useful in detecting the DAIs seen in mTBI; more specific measures of EEG associated with cognitive processes, such as event-related potentials, may be better at detecting the attention and memory deficits related to mTBI.^63^ MEG, in conjunction with MRI, has also been shown to be useful in detecting abnormal activity in patients with PCS.^64^

Despite the multitude of available imaging and behavioral assessments of mTBI, there lacks a cohesive neurobiological explanation for the cognitive deficits observed. To understand the spectrum of mTBI outcome, we recommend an approach that unifies anatomical and behavioral assessments.

## A unified approach to mTBI

### Anatomy: diffusion tensor imaging

Unlike the traditional CT and MRI, DTI is an MRI modality that can provide quantitative characterization of intrinsic features of tissue microstructure and microdynamics.^65^ DTI has provided a powerful new tool for detecting DAI and other microstructural changes in white matter associated with mTBI injury severity.^5–8^ Although still considered experimental, the application of DTI shows great potential for the clinical diagnosis of mTBI.^52,66^

The three principle eigenvalues of the diffusion tensor matrix quantitatively describe the mobility of water molecules. Axial diffusivity is the largest of the eigenvalues and represents molecular mobility parallel to the local fiber tract direction; radial diffusivity is the average of the other two principle eigenvalues and represents mobility perpendicular to the fiber tract direction. Mean diffusivity serves as an index of water molecule mobility averaged over all directions. Changes in the axial and radial diffusivity indices may be used to specify the pathology that leads to changes in diffusion anisotropy, for example, myelin loss or axonal injury.^67^

Of the several quantitative parameters that can be derived from DTI,^65^ fractional anisotropy (FA) is considered to be a robust indicator of white matter microstructural integrity.^5–8,27,53,68–70^ In a parallel fiber arrangement of a white matter tract, the diffusion of water molecules is directionally constrained, resulting in a high FA value. The theoretical range of FA values is from 0 (isotropic) to 1 (completely anisotropic); the larger the value of the index, the larger the water molecule directionality. Either an increase above or decrease below the normal FA range likely indicates white matter abnormality.

Changes in axial diffusivity, radial diffusivity, and FA, may indicate different types of white matter abnormality, which may reflect different phases of progression of TBI.^66,71^ Although there are still issues to be addressed, the DTI technology has so far demonstrated conclusively that mTBI is associated with wide-spread structural changes in cortical white matter tracts.^5–8,27,53,68–70,72^ Also of note, the quantitative nature of DTI provides the opportunity to correlate injury severity with functional deficits measured by neuropsychological tests and other behavioral measures.^8,53,70,73^

### Behavior: symptom assessment

The functional deficits associated with mTBI can be accounted by microstructural changes in the frontal white matter. Frequently, the outcome of DAI is strikingly similar to that of focal damage in the frontal lobe.^74^ The cognitive symptoms of both types of injuries include deficits associated with concentration, memory, and high-level executive functions, such as planning and decision making.^54,57,74,75^

It has been suggested that preparatory neural activity, i.e., attention, enables the efficient integration of sensory information and goal-based representations.^76^ This theoretical framework allows PCS to be grouped into primary and secondary symptoms (Table 2). Primary symptoms are suggested to arise directly from the physical brain injury, which is suspected to occur in the white matter tracts that connect the prefrontal–cerebellar network. The result of this disruption is considered to be impairment in predictive timing, which causes increased distractibility (attention deficits), working memory deficits, and problems with balance and coordination: symptoms commonly displayed by mTBI patients. An inability to correctly time or anticipate sensory events would result in a temporal mismatch of sensory expectation to actual sensory input, potentially leading to dizziness, tinnitus, and sensory hypersensitivity. Secondary symptoms could arise from increased activation of the prefrontal cortex (PFC), which might occur because of an increase in error signals and performance variability. Serving as a compensatory mechanism, the PFC could be recruited to help bridge the moment-to-moment temporal discrepancies. This increased effort might underlie fatigue, headache, irritability, anxiety, and when prolonged, depression.

**Table 2:** Attention-based categorization of postconcussive symptoms

Primary symptoms related to predictive timing deficit	Secondary symptoms related to PFC compensation and error signaling
• Decreased concentration	• Headache
• Memory difficulties	• Fatigue
• Decreased processing speed	• Sleep disturbances
• Decreased awareness	• Irritability
• Balance and coordination problems	• Depression• Anxiety
• Blurred vision	
• Dizziness	
• Tinnitus	
• Hypersensitivity to light or noise	

Adapted from Ghajar and Ivry.^76^

### Behavior: eye tracking

Frequent lapses in attention are a characteristic symptom of TBI.^77^ Traditional measures that use discrete responses are unable to detect momentary lapses in attention. This limitation may contribute to the relative insensitivity of neuropsychological tests in detecting mTBI.^1,2,50,51^ Anti-saccades tasks, a type of eye-movement paradigm sensitive to frontal lobe dysfunction,^78^ rely on discrete stimulus-response sets. Anti-saccades tasks may be useful once subjects have perceived PCS symptoms^79^; however, this paradigm may not be sensitive to acute mTBI.^80^ Because attention varies over time, a relatively continuous measure of performance is needed to detect moment-to-moment fluctuations in attention within individuals.^76^

The examination of performance of visual tracking of a moving target may provide a supplement to conventional behavioral assessments of mTBI patients.^79,81^ Using video-oculography, eye movement can be monitored easily, precisely, and continuously. In contrast to the anti-saccade paradigm, visual tracking does not rely on discrete stimulus-response sets during the maintenance phase. Visual tracking of a moving target requires the integration of multiple sensory inputs and one's own motor efforts.^82^ Visual tracking also requires cognitive processes including target selection, sustenance of attention, spatio-temporal memory, and expectation.^83–85^

Quantification of visual tracking performance using a circular target trajectory^86,87^ provides a continuous behavioral assessment metric. The motion of a target traveling at a constant velocity with a fixed radius from the center is highly predictable. This movement can continue indefinitely within the orbital range of the eye, which makes the stimulus particularly suitable for studying the processes required to maintain predictive visual tracking. Predictive visual tracking requires both attention and working memory,^85^ processes for which the PFC is considered to be an important substrate.^88^ These cognitive functions are often compromised in mTBI patients.^17,89^

**Figure 1:**
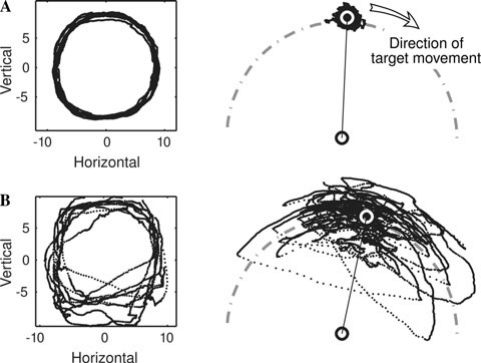
Visual tracking of a target moving in a circular trajectory of 8.5° radius at 0.4 Hz. (**A**) Example of a good performance by a normal subject. (**B**) Example of a poor performance by a subject with chronic postconcussive symptoms. Right panel: Two-dimensional trajectory of the gaze superimposed over nine cycles. Left panel: Scattergram of gaze positions relative to the target fixed at the 12 o'clock position. The white circle indicates the average gaze position. The dot–dashed curve indicates the circular path.

Visual tracking performance can be objectively quantified using parameters, such as smooth pursuit velocity gain, phase error, and root-mean-square error. Because TBI is known to increase intra-individual performance variability on visuo-motor tasks,^77,90^ we measured the variability of visual tracking performance in terms of gaze positional error relative to the target to grade the level of performance.^81^ Good visual tracking was characterized by overall tight clustering of the gaze positions around the target (Fig. 1A). In contrast, poor visual tracking in mTBI subjects was generally characterized by a wide distribution of the gaze along the circular path, which indicates spatio-temporal dyssynchrony with the stimulus (Fig. 1B). The spread of visual-tracking gaze errors (variability) can be used as an attention metric and can be correlated with an individual's degree of white matter integrity.

### Eye-tracking and DTI

**Figure 2:**
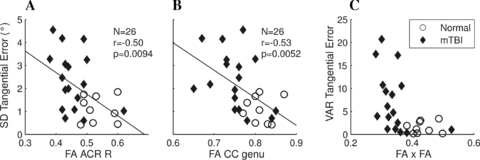
Relationships between FA values and visual tracking performance variability in the tangential direction of the target trajectory. (**A**) Right ACR. (**B**) Genu of the corpus callosum (CC). The regression lines were determined from the combined subject population. (**C**) Cross-factorization of A and B (multiplication of respective abscissa and ordinates). Circles, normal subjects; Diamonds, subjects with chronic postconcussive symptoms; SD, standard deviation; VAR, variance.

We quantified visual-tracking gaze error variability by the standard deviation of the error distribution and compared this parameter to DTI FA values.^81^ Large gaze error variability was associated with low mean FA values in the right anterior corona radiata (ACR; Fig. 2A), the left superior cerebellar peduncle (not shown) and the genu of the corpus callosum (Fig. 2B). The right ACR and left superior cerebellar peduncle are tracts known to support the sustenance of attention and spatial processing.^88,91–93^ Both the ACR and genu include fibers connecting to the dorsolateral PFC (DLPFC).^94^ Clustering of the normal and mTBI subject populations is observed along both the DTI FA and gaze variability axes (Fig. 2A and B). Because the ACR and the genu are among the most frequently damaged white matter tracts in mTBI,^53^ the correlations imply that gaze error variability during visual tracking may provide a useful screening tool for mTBI. When the FA values of the right ACR and the genu are cross-factored, the graph tends to further dissociate the normal and mTBI subject populations (Fig. 2C).

The right DLPFC may be particularly significant to mTBI symptomatology. It is a central site in the synthesis of diverse information needed to carry out complex behaviors^88^ and also serves as a node in the attention network.^95^ We postulate that increased visual tracking variability is a consequence of the dyssynchrony between moment-to-moment expectations and incoming sensory input, caused by deficits in the right prefrontal–left cerebellar loop. Given the vulnerability of the frontal white matter to mTBI, the degree to which the connection to the right DLPFC is damaged by the injury may be estimated by visual tracking variability. The function of the DLPFC is also considered to be a convergent factor between posttraumatic stress disorder and persistent PCS.^39^ As such, the presence and extent of damage in the right prefrontal cortical connection could potentially serve during the acute stages of mTBI as a predictor of which mTBI patients will develop chronic symptoms.^81^

Individual differences in the outcome of mTBI may be predicted by identifiable risk factors,^39^ and therefore it would be useful to measure performance on a predictive visual tracking task acutely after mTBI to compare with longitudinal results. Also, as structural connectivity may be improved by behavioral training,^96^ a biofeedback paradigm to improve visual tracking performance could potentially be used therapeutically.

## Conclusion

Predictive visual tracking shows promise as an attention metric to assess severity of mTBI. Deficits seen during predictive visual tracking correlate with DTI findings and with observed damage to neural pathways known to carry out cognitive and affective functions that are vulnerable to mTBI. The paradigm we have developed for testing subjects is currently under 5 min in duration for the entire test, which is markedly shorter compared to most neuropsychological tests.

In summary, the approach presented in this paper unifies the anatomical and behavioral deficits characteristic of mTBI, and allows for the design and deployment of preventative, diagnostic, and therapeutic interventions that will improve the outcome of mTBI patients.
